# refloraR: An R package for efficiently retrieving and analyzing plant specimen data from the Herbário Virtual Reflora

**DOI:** 10.1002/aps3.70081

**Published:** 2026-09-16

**Authors:** Carlos Calderón del Cid, Ana Flávia Alves Versiane, Paula Leitman, Fabiana Ranzato Filardi, Rafaela Campostrini Forzza, Domingos Cardoso

**Affiliations:** ^1^ Universidade de São Paulo São Paulo São Paulo Brazil; ^2^ Jardim Botânico do Rio de Janeiro Rio de Janeiro Rio de Janeiro Brazil; ^3^ Instituto Chico Mendes de Conservação da Biodiversidade, Parque Nacional do Descobrimento Prado Bahia Brazil

**Keywords:** biodiversity informatics, Brazilian flora, herbarium collections

## Abstract

**Premise:**

Advances in the digitization of herbarium collections are enabling open access to specimen data for research and conservation. In Brazil, the Herbário Virtual Reflora (HVR) hosts over 4.8 million high‐resolution images of botanical specimens and their associated data from 86 national and international herbaria.

**Methods and Results:**

To facilitate access and use of HVR data in biodiversity research, we developed the R package refloraR. This tool interacts with the HVR Integrated Publishing Toolkit (IPT) to download, parse, summarize, and filter herbarium records in Darwin Core Archive (DwC‐A) and dataframes. It also retrieves occurrence records and indeterminate specimens for any hierarchical level. We provide a working example that combines the refloraR functions for quantifying indeterminate specimens as an indicator of taxonomic gaps within and among collections.

**Conclusions:**

The refloraR package supports reproducible workflows in systematics, conservation, and ecology. By enabling the efficient retrieval of specimen data, it integrates the HVR into modern biodiversity informatics and democratizes access to natural history collections.

## Resumo

**Premissa:**

Avanços na digitalização dos herbários viabilizam o acesso aberto aos dados de espécimes para pesquisa e conservação. No Brasil, o Herbário Virtual Reflora (HVR) hospeda mais de 4,8 milhões de imagens em alta resolução de amostras botânicas e seus dados associados provenientes de 86 herbários nacionais e estrangeiros.

**Métodos e Resultados:**

Para facilitar o acesso e uso dos dados do HVR em pesquisas de biodiversidade, desenvolvemos o pacote R refloraR. Esta ferramenta interage com o Integrated Publishing Toolkit (IPT) do HVR para baixar, processar, resumir e filtrar registros de herbário no formato Darwin Core Archive (DwC‐A). Também recupera registros de ocorrência e de espécimes indeterminados para qualquer nível hierárquico. Apresentamos um exemplo prático, combinando as funções do refloraR para quantificar espécimes indeterminados como indicadores de lacunas taxonômicas nas coleções.

**Conclusões:**

O refloraR apoia fluxos de trabalho reprodutíveis em sistemática, conservação e ecologia. Ao permitir a recuperação eficiente de dados de espécimes, integra o HVR à informática moderna sobre biodiversidade e democratiza o acesso às coleções de história natural.

The Anthropocene is marked by global environmental changes in Earth's biogeochemical cycles, biodiversity, and ecosystems due to human activities (Lewis and Maslin, 2015). These environmental impacts, coupled with advances in digital infrastructure, have heightened the urgency of developing tools to streamline access to large‐scale data from natural history collections (NHC) and enhance our understanding of biodiverse systems (Nelson and Ellis, 2018). NHCs encompass natural history museums and herbaria, digital databases of specimen‐derived material, and living collections housing invertebrates, vertebrates, fungi, plants, and bacteria (Vollmar et al., 2010; Miller et al., 2020). The taxonomic, geographic, and temporal richness of NHCs provides essential insights into both the modern and historical natural world, enabling investigations into biotic responses to human activity and broader evolutionary processes (Holmes et al., 2016; Hedrick et al., 2020; Miller et al., 2020). The digitization of NHCs has emerged as a cornerstone in modern biodiversity science, enhancing the longevity of physical specimens by minimizing handling (Whittaker and Phillips, 2024) and drastically increasing accessibility for researchers worldwide (Martellos and Seggi, 2024). It also supports downstream users such as the general public, policymakers, conservation organizations, and private enterprises (Beaman and Cellinese, 2012).

In this context, herbarium collections serve as invaluable repositories of plant and fungal biodiversity data, spanning international to regional scales (Mandrioli, 2023). Globally, more than 3500 herbaria house approximately 396 million botanical specimens (Index Herbariorum, 2026). These collections are instrumental in systematics, ecology, and conservation (Canteiro et al., 2019) and act as historical archives documenting global environmental transformations over centuries (Shweta et al., 2026). Innovations in specimen imaging (e.g., Jabot image and Jabot transfer; Versiane et al., 2026), automated text capture (Guralnick et al., 2025; Turnbull et al., 2025), and digitization workflows (Nelson et al., 2015; Thompson and Birch, 2023) have facilitated the digitization of herbaria. This effort has become a global priority, enabling open access to specimens that were once limited to a few institutions or experts (Soltis, 2017).

Recent years have seen a surge in novel applications of herbarium data in biodiversity science (Vasconcelos and Boyko, 2025). To fully leverage the digitized collections, computational tools are needed to access, process, clean, and analyze specimen data efficiently. Such tools are essential to overcoming the common limitations of biological datasets while maximizing the research value of botanical collections (Zuanny et al., 2024). Online herbarium databases foster information sharing, encourage cross‐institutional collaboration, and support workforce development in biodiversity informatics, image services, and geospatial technologies (Harris and Marsico, 2017).

In Brazil, the Reflora program (http://reflora.jbrj.gov.br/), launched in 2010, has advanced the digitization and open dissemination of Brazilian plant specimens housed in herbaria worldwide. Through the Herbário Virtual Reflora (HVR; https://reflora.jbrj.gov.br/reflora/herbarioVirtual), developed by the Jardim Botânico do Rio de Janeiro (JBRJ) in collaboration with COPPE/Universidade Federal do Rio de Janeiro (UFRJ), the Reflora program now provides open access to high‐resolution images and associated specimen metadata from 86 national and international herbaria, representing one of the largest coordinated digitization efforts globally (BFG [The Brazil Flora Group], 2022; Pinheiro et al., 2024; Reflora, 2025; Versiane et al., 2026). HVR is integrated with Flora e Funga do Brasil (FFB; https://reflora.jbrj.gov.br/consulta/), linking voucher specimens to taxonomic accounts, supporting conservation assessments by the Centro Nacional de Conservação da Flora (CNCFlora; https://cncflora.jbrj.gov.br/), and enabling registered taxonomists to curate and verify records remotely (Versiane et al., 2026). All records are published via the HVR Integrated Publishing Toolkit (IPT; https://ipt.jbrj.gov.br/reflora), ensuring open access and data transparency. As of 2025, HVR hosts over 4.8 million specimen images and their associated collection information.

Here, we introduce the refloraR package, a set of R functions (R Core Team, 2025) that enable streamlined access to Reflora data. The package interacts directly with the HVR IPT, allowing users to download complete specimen datasets in Darwin Core Archive (DwC‐A) format. Additionally, it includes tools to summarize collection metadata and filter records based on taxonomic rank, spatial distribution, or data completeness. The package was designed to support both research and institutional curation by simplifying data acquisition and enabling reproducible workflows using specimen‐derived data.

A recent complementary tool in the Brazilian biodiversity data ecosystem is the catalogoUCsBR package, which focuses on facilitating checklist construction for protected areas (Bochorny et al., 2025). This R package and its Shiny web interface enable users to combine herbarium records from multiple sources, retrieve accepted species names, and prepare curated lists for the Catálogo de Plantas das Unidades de Conservação do Brasil (https://catalogo-ucs-brasil.jbrj.gov.br/). Like refloraR, it is designed to improve data usability and reproducibility. Together, these tools highlight the growing role of open‐source computational resources in transforming access to Brazil's rich botanical heritage and facilitating conservation decisions that are grounded in up‐to‐date, taxonomically vetted information.

## METHODS AND RESULTS

The refloraR package is an open‐source R toolkit designed to streamline the access, management, and analysis of plant specimen data from the HVR, hosted by JBRJ. The current version of refloraR is available on GitHub (https://github.com/DBOSlab/refloraR; see Data Availability Statement).

**Figure 1:**
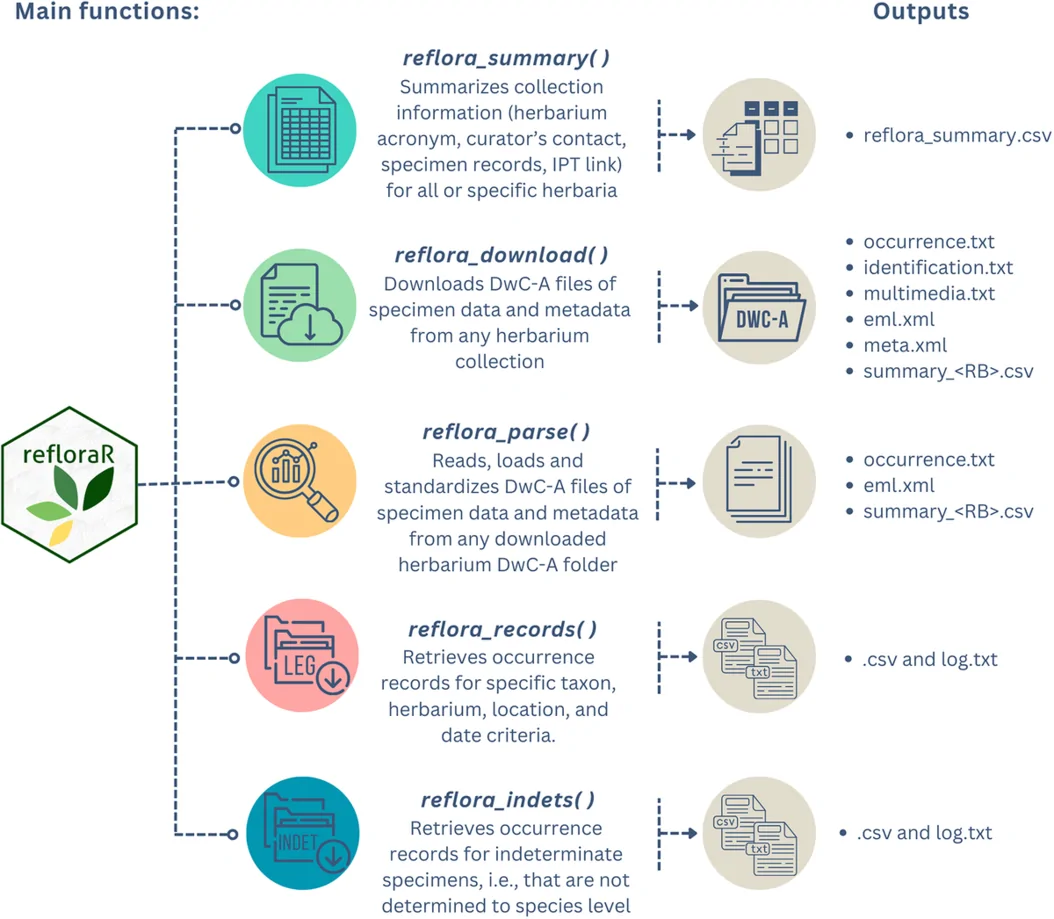
Overview of the main functions available in the refloraR package, along with their expected outputs. Each function is designed to support access, filtering, and processing of plant specimen data from the Herbário Virtual Reflora (HVR), hosted by the Jardim Botânico do Rio de Janeiro (JBRJ). DwC‐A, Darwin Core Archive.

**Figure 2:**
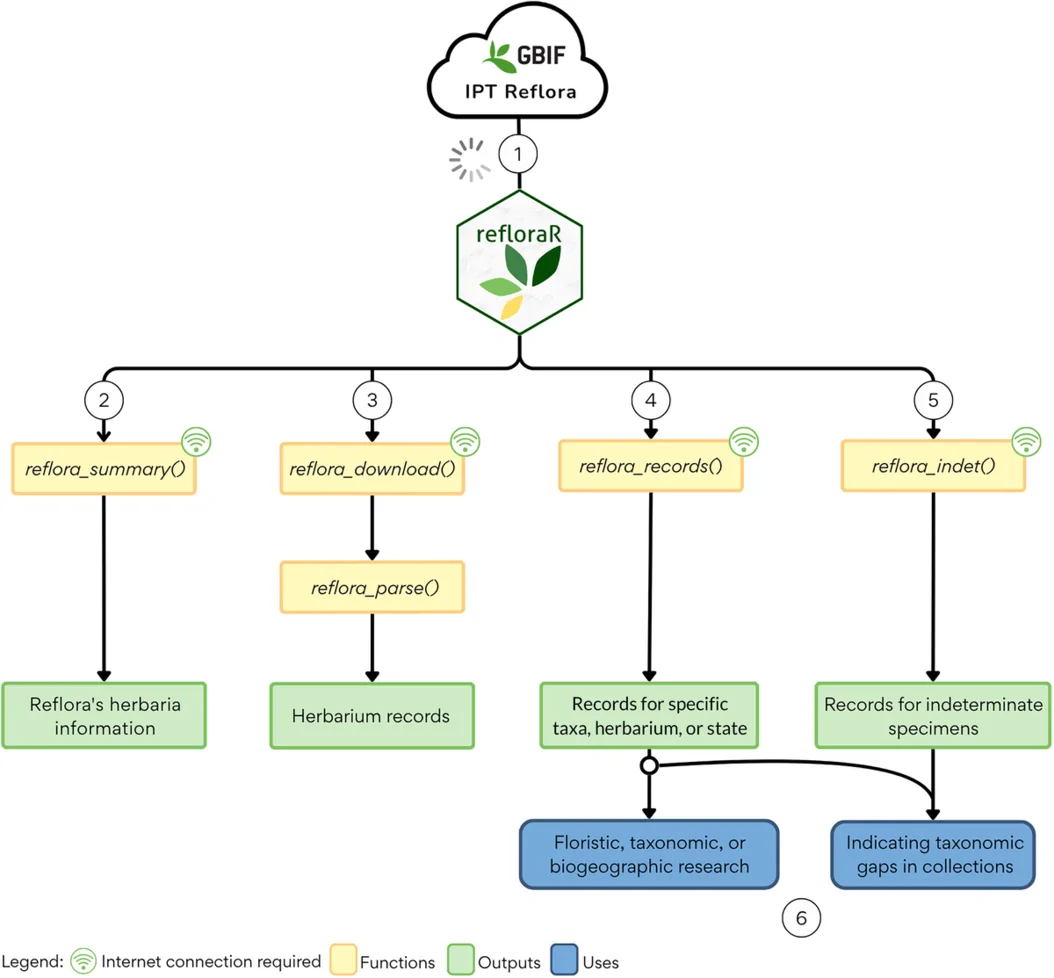
Workflow for retrieving and processing data using the refloraR package. (1) It starts by querying available collections through the Reflora Integrated Publishing Toolkit (IPT) node hosted by the Global Biodiversity Information Facility (GBIF). (2) *reflora_summary()* inspects available collections and metadata. (3) Users can download Darwin Core Archive (DwC‐A) files for selected herbaria using *reflora_download()*; these files are parsed and standardized locally using *reflora_parse()*. (4) *reflora_records()* retrieves occurrence records based on taxonomic, geographic, and temporal criteria. (5) *reflora_indets()* extracts occurrence data for indeterminate specimens (e.g., identified only to family or genus level). (6) Final outputs can be used to support floristic, taxonomic, or biogeographic research or as an indicator of taxonomic gaps within and among collections. The package requires an internet connection only for initial queries and downloading/updating herbarium data. All other functions can operate offline if data have been previously downloaded.

Comprehensive documentation and step‐by‐step articles are available at the package's official website (https://dboslab.github.io/refloraR-website/). The refloraR package is organized into five key processing categories: (1) summarizing collections, (2) downloading records, (3) parsing DwC‐A files, and (4 and 5) retrieving specimen records, either specific (4) or indeterminate (5) specimens. These categories are implemented through five core functions of the refloraR package (Table 1, Figures 1 and 2).

1. **Summary and metadata exploration**: The function *reflora_summary()* provides a summary of available plant specimen records from all herbaria published in the Reflora network. This function generates a tidy dataframe that includes total record counts and key IPT metadata (e.g., publication version/date, contact information, and a direct link to each collection's IPT resource). Importantly, the summary also reports each collection's repatriation status, distinguishing digitally repatriated collections—Brazilian specimens physically housed in foreign herbaria but made openly accessible through Reflora via high‐resolution images and associated metadata—from other published collections, thereby supporting taxonomic revision, curation, and national biodiversity assessments in Brazil.
2. **Data download:** The package uses the function *reflora_download()* to enable the bulk download of specimen records for one or multiple herbaria from the Reflora IPT. Downloads are structured in DwC‐A format, organized in local folders, and ready for parsing. Internet access is required only for initial queries, downloads, or updates.
3. **Data parsing and standardization:** The function *reflora_parse()* converts Reflora DwC‐A downloads into structured R objects and returns a list with (i) occurrence data, (ii) dataset‐level metadata, and (iii) repository‐level summaries. Parsing applies conservative, light‐touch cleaning to improve internal consistency while preserving original fields; this cleaning includes: (a) fixes common field misplacements (e.g., family names entered in the genus column), (b) standardizes taxonRank to the Global Biodiversity Information Facility (GBIF)/Darwin Core controlled vocabulary (family, genus, species, etc.), (c) harmonizes column names and basic formats for downstream processing, (d) corrects obvious typographical errors in family names using a curated set of regular‐expression patterns that cover common orthographic variants and misspellings.
  Large, aggregated herbarium datasets frequently contain misidentifications, outdated names, typographical errors, synonym inflation, and cultivated or extra‐biome taxa. Goodwin et al. (2015) highlight that a substantial fraction of tropical specimens are misnamed or not updated to current taxonomy, and Cardoso et al. (2017) show how unchecked aggregation can inflate diversity estimates (e.g., duplicate counts under long‐deprecated synonyms, repeated listings of common species under multiple names) in Amazonian tree checklists. In light of these issues, *reflora_parse()* is deliberately minimal to preserve provenance, relying on a separate, stricter standardization workflow that performs curated name reconciliation. This design enables robust downstream analyses and avoids the pitfalls of indiscriminate database aggregation in tropical systems. For full taxonomic normalization (accepted names, synonym resolution, and rank/placement updates across families and genera), we recommend the use of a dedicated taxonomic standardization function provided by the recently published floraBR package (Trindade, 2024). This step is strongly recommended prior to ecological, macroevolutionary, or biodiversity‐informatics analyses, rather than working directly from raw Reflora‐IPT records.
4. **Taxon‐based record retrieval:** The function *reflora_records()* automates the search and retrieval of specimen records for specific taxa. It combines downloading, parsing, filtering, and optional file export. It outputs a dataframe of matched records and a session log (if save = TRUE). All retrieved records include direct URLs to specimen images hosted at HVR (column “bibliographicCitation”) and links to download the original high‐resolution images (column “associatedMedia”).
5. **Indeterminate record retrieval:** With the function *reflora_indets()*, it is possible to specifically retrieve indeterminate specimen records, defined as those identified only to the family or genus level. This function includes filters for taxon, herbarium, state, and collection year. The output is a filtered dataframe of records, with optional CSV export and a log file summarizing record counts by herbarium, family, genus, country, and state/province.

**Table 1:** Overview of the main functions in the refloraR package for streamlined access to the Herbário Virtual Reflora (HVR) in the R environment.

Function name	Description	Output
*reflora_summary()* a	Summarizes the current available plant specimen records from the HVR.	A dataframe with one row per herbarium collection, summarizing key institutional data and metadata.
*reflora_download()* a	Downloads full DwC‐A files from any or all Reflora herbaria.	A local folder with DwC‐A files for a specific herbarium or all Reflora‐associated herbaria.
*reflora_parse()*	Reads and standardizes DwC‐A files from downloaded archives into structured data usable in R.	A named list with parsed data tables, metadata, and IPT descriptors.
*reflora_records()* a	Retrieves filtered occurrence records for one or more taxa. Handles automatic download and parsing.	A dataframe of filtered occurrences, including URLs to specimen images and high‐resolution downloads. Optional export to CSV and log file documenting the session.
*reflora_indets()* a	Retrieves records for indeterminate specimens (i.e., those identified only to family or genus level), with optional filters (e.g., taxon, state).	A dataframe with indeterminate records, including direct links to images and high‐resolution files. Optional export to CSV and log file with summary statistics and collection breakdowns.

*Note*: DwC‐A, Darwin Core Archive; IPT, Integrated Publishing Toolkit.

^a^ Requires an internet connection to run.

Importantly, once the data are downloaded, all subsequent functions (e.g., *reflora_parse()*, *reflora_indets()*) can be run offline. This supports robust, reproducible workflows during fieldwork and in environments with limited connectivity. While refloraR provides basic data cleaning and standardization, we recommend combining its outputs with post‐processing tools such as bdc (Ribeiro et al., 2022), coordinatecleaner (Zizka et al., 2019), and barRoso (https://dboslab.github.io/barRoso-website/) for rigorous data curation, particularly when records are used in spatial modeling or conservation assessments. The main functions and their respective outputs of the refloraR package are summarized in Table 1.

### Working example: Mapping indeterminate specimens from the Herbário Virtual Reflora

A key advantage of the refloraR package is its ability to summarize and filter large‐scale herbarium datasets to support targeted taxonomic work. One illustrative application is to map and quantify indeterminate specimens (i.e., records identified only to family or genus) as an operational indicator of unresolved identifications and potential taxonomic knowledge gaps within and among collections. For this purpose, the package provides the function *reflora_indets()*, which retrieves occurrence records of indeterminate specimens from the HVR. These outputs can be used to compare the frequency and distribution of indeterminate specimens across herbaria and taxonomic groups, helping to identify collections with unusually high numbers of unresolved records, to highlight taxa and regions where additional identification effort may be most needed, to point out taxonomic groups that require a taxonomist for their study, and to flag taxonomic groups that may require targeted specialist attention.

We note that some poorly known or undescribed species may be misidentified rather than left indeterminate. Therefore, *reflora_indets()* should be interpreted as an exploratory prioritization tool rather than a comprehensive assessment of taxonomic knowledge gaps. It allows researchers to: (1) visualize the number and proportion of indeterminate specimens across HVR's associated collections; (2) prioritize taxonomic work on understudied groups with high rates of indeterminate material; and (3) highlight herbaria that curate large volumes of unidentified or taxonomically unresolved records (e.g., specimens not identified to species level, including records identified only to family or genus), which may represent overlooked or undescribed taxa.

**Figure 3:**
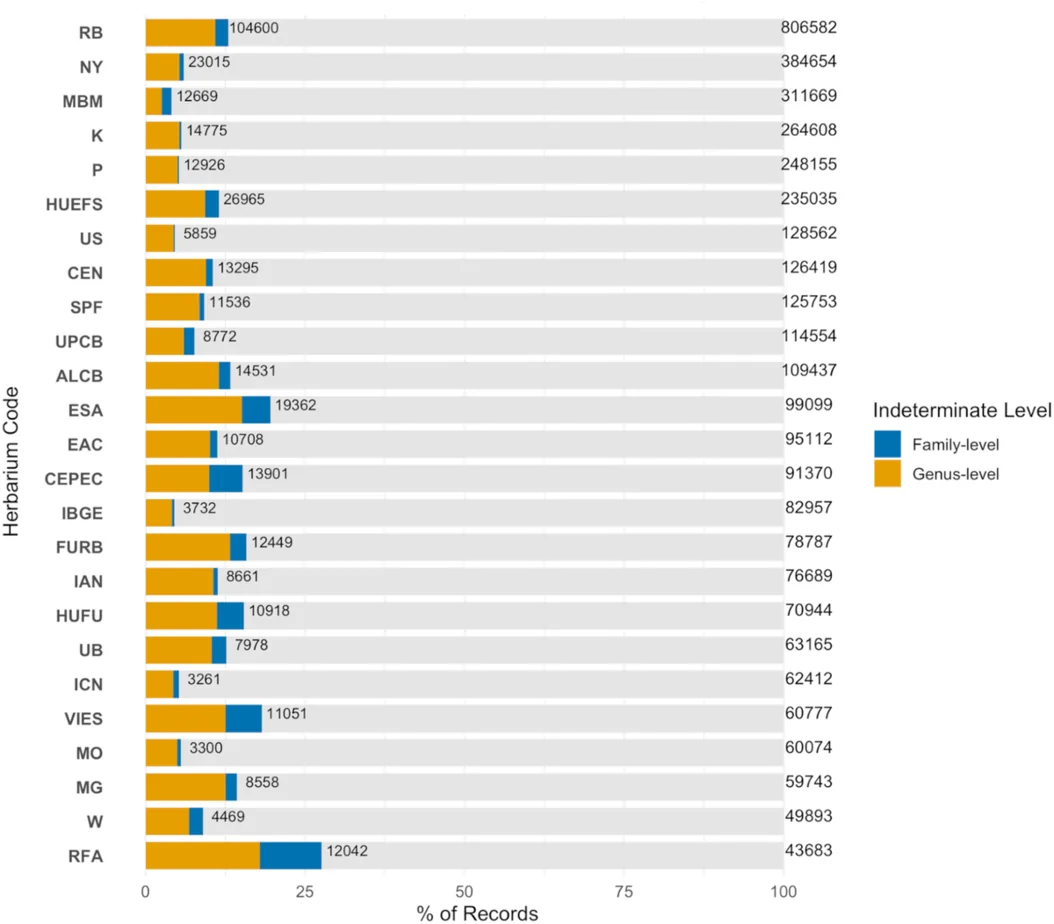
Proportion of indeterminate specimens among the 25 most record‐rich herbaria in the Herbário Virtual Reflora (HVR). Colored segments show, for each herbarium, the percentage of records identified only to genus (orange) or family (dark blue). The light gray background bar indicates the total number of records per herbarium (100%). Numbers beside the colored segments give the total indeterminate count (genus + family), and numbers at the end of the gray bars indicate the total number of records per herbarium within the HVR database. Herbarium acronyms per Index Herbariorum (2026).

**Figure 4:**
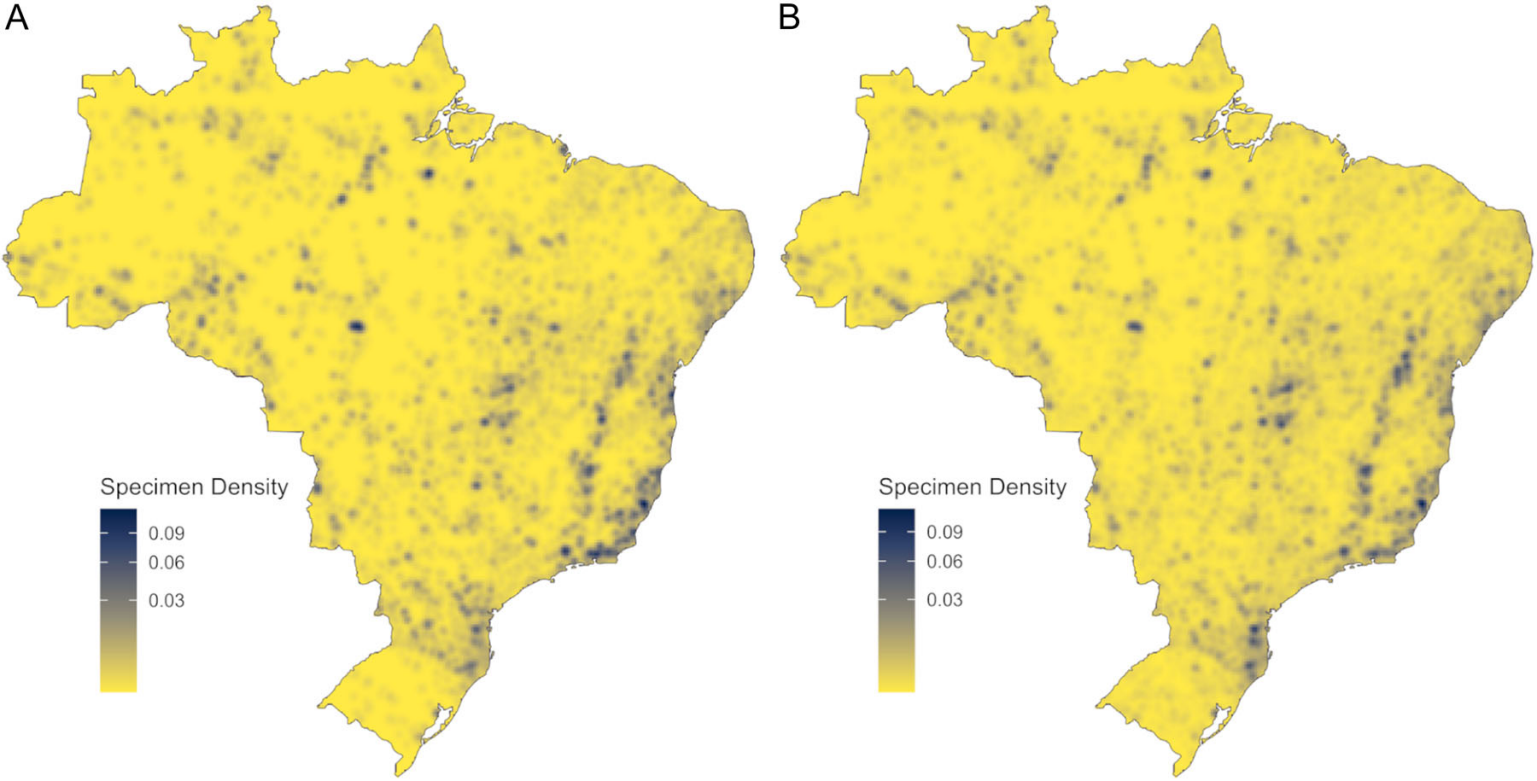
Spatial density of indeterminate plant specimens collected across Brazil and available in Herbário Virtual Reflora (HVR): identified to family level only (A) and identified to genus level only (B). The color gradient represents the density of collected but taxonomically unresolved specimens, with the gradient ranging from dark blue (high density) to yellow (low density).

In the example provided here (Appendix S1; see Supporting Information with this article), we used *reflora_indets()* to retrieve all indeterminate records currently available in HVR, restricting the search to occurrences from Brazil. Basic geographic filtering was applied to exclude duplicate specimens using the barRoso package (https://dboslab.github.io/barRoso-website/) and records with missing or invalid coordinates using coordinatecleaner (Zizka et al., 2019). We then visualized two outputs: first, a bar plot showing the proportion of indeterminate specimens among the top 25 herbaria ranked by total number of indeterminate records, categorized by taxonomic rank (identified only to family or genus level; Figure 3); and second, a pair of smoothed raster maps showing the spatial distribution of indeterminate specimens across Brazil, based on specimen density (Figure 4).

We observe that herbaria with the largest collections (e.g., RB, NY, and HUEFS; herbarium acronyms per Index Herbariorum [2026]) also hold the highest absolute counts of indeterminate specimens, but this effect does not reflect proportionally to their collection size (Figure 3). In contrast, some herbaria with smaller collections (e.g., RON, COR, RFA) exhibit relatively high proportions of unresolved records. Across all herbaria, the majority of indeterminacy occurs at the genus level rather than the family level. Therefore, smaller regional herbaria may represent critical targets for taxonomic revision, as they contribute disproportionately high shares of unidentified specimens relative to their total collections. This observation aligns with recent findings by Delves et al. (2024), who demonstrated that small and often under‐resourced herbaria in megadiverse countries provide essential, unique data that improve the accuracy of conservation assessments and are indispensable for achieving international biodiversity and conservation targets.

The spatial distribution of indeterminate specimens across Brazil (Figure 4) is broadly similar between the maps for specimens identified to the family and the genus level. In both cases, the highest densities of unresolved records are clustered along the biodiversity‐rich Atlantic coast and the Espinhaço Range. This pattern likely reflects, in part, the greater sampling intensity historically associated with southeastern Brazil (Zorzetto et al., 2006). The region concentrates numerous research institutions and active botanical expeditions, which generate large volumes of material requiring taxonomic determination. Likewise, major herbaria in this region are frequently national reference centers, receiving specimens collected from multiple parts of the country or abroad, for specialists’ curation and identification. For this reason, these institutions store a substantial volume of material awaiting determination. Despite having more specialists and infrastructure, the incoming specimens can exceed the available curatorial capacity, generating significant records unresolved at the species level. Therefore, high numbers of indeterminate specimens in southeastern Brazil may represent centralized curatorial dynamics, rather than a lack of expertise.

In contrast, vast areas of the Amazon basin, despite being among the most biodiverse regions on the planet (Cardoso et al., 2017), display significantly lower densities of indeterminate specimens, excepting a collection hub in Mato Grosso. Importantly, these low numbers of indeterminate specimens do not necessarily imply greater taxonomic resolution in the Amazon, but rather a lack of sampling intensity and available expertise (Brito, 2010).

This workflow highlights the value of refloraR as a diagnostic tool to support taxonomic research and herbarium curation. Indeterminate specimens, often underutilized in biodiversity studies, may contain valuable information, including potential new species (Bebber et al., 2010). By identifying these data gaps and their institutional or spatial distribution, researchers can more effectively target efforts to advance biodiversity documentation in Brazil.

## CONCLUSIONS

The development of the refloraR package represents a significant step toward enhancing the accessibility, usability, and analytical potential of herbarium specimen data. By providing a set of user‐friendly functions for downloading, parsing, summarizing, and filtering records from the HVR, the package enables researchers and curators to build reproducible workflows directly within the R environment. Importantly, refloraR supports both full data integration for taxonomic and ecological research as well as targeted queries, such as retrieving indeterminate specimens, to diagnose data gaps and address long‐standing knowledge shortfalls. The inclusion of minimal but effective data standardization further reduces entry barriers for researchers unfamiliar with biodiversity data standards. Furthermore, all records retrieved through refloraR include direct links to their corresponding specimen images, ensuring that taxonomic verification and data use remain traceable to the original digital vouchers.

Moreover, by promoting the use of Reflora data in conjunction with other R tools for cleaning and geospatial validation (e.g., coordinatecleaner and barRoso), refloraR fits into a growing ecosystem of reproducible biodiversity informatics pipelines. This is particularly critical for Brazil, a megadiverse country whose botanical data resources are now becoming more open, structured, and actionable. Ultimately, refloraR contributes to a broader agenda of democratizing access to biological collections, promoting taxonomic transparency, and enabling researchers to make more effective use of digital herbarium infrastructure. As the availability of digitized specimens and herbarium data continues to grow, tools like refloraR will be essential for realizing the scientific and conservation potential of these invaluable resources.

The future development of the refloraR package will focus on broadening functionality and deepening integration with other biodiversity informatics tools. Planned improvements include enhanced internal functions for data standardization, as well as taxonomic resolution and reconciliation using services such as GBIF's Species Matching API and the Flora e Funga do Brasil (https://reflora.jbrj.gov.br/consulta/) checklist to validate and correct scientific names. Beyond technical enhancements, the long‐term vision of refloraR is to empower Brazilian and global users to harness Reflora program data in support of open, reproducible, and impactful botanical science.

## AUTHOR CONTRIBUTIONS

D.C. and C.C.d.C. planned the research and developed and designed the package. D.C. wrote the package website, including the how‐to guides. C.C.d.C., A.F.A.V., and D.C. wrote the initial draft of the manuscript and prepared the figures. All authors contributed to and approved the final version of the manuscript.

## Supporting information


**Appendix S1:** R code with a full description of the pipeline.

## Data Availability

The most recent development version of the refloraR package is freely available at: https://github.com/DBOSlab/refloraR. Complete documentation, tutorials, and function‐specific guidance are hosted at: https://dboslab.github.io/refloraR-website/. All code used to generate the figures and the working example in this paper is available in the Supporting Information. All specimen data used in this study were obtained from the Herbário Virtual Reflora (HVR) via the refloraR package. The package accesses openly licensed data published through the HVR Integrated Publishing Toolkit (IPT), maintained by the Jardim Botânico do Rio de Janeiro (JBRJ).
