# Supplementary material for: refloraR: An R package for efficiently retrieving and analyzing plant specimen data from the Herbário Virtual Reflora
Source: Appl Plant Sci. 2026 Sep 16:e70081. Online ahead of print. doi: 10.1002/aps3.70081 (PMC13583462; doi:10.1002/aps3.70081)
Supplement: Supplementary file 1 [file APS3-9999-e70081-s001.html]

Analyzing Indeterminate Herbarium Specimens in Brazil


# Analyzing Indeterminate Herbarium Specimens in Brazil

#### Carlos Calderón del Cid/Domingos Cardoso

# Introduction

This tutorial demonstrates how to analyze indeterminate plant
specimens (i.e., records only identified to family or genus level) from
the Reflora
Virtual Herbarium using the `refloraR` package.
Particullarly, we will be using the `reflora_indets()`
function. We will visualize the indeterminate specimens across the top
25 herbaria and map the spatial distribution of low-resolution records,
identified only to genus and family level.

# Load Required Packages

```
library(tidyverse)
library(maps)
library(patchwork)
library(forcats)
library(sf)
library(letsR)
library(CoordinateCleaner)
library(geobr)
library(ggplot2)
library(dplyr)
library(tidyr)
library(rnaturalearth)
library(rnaturalearthdata)
library(viridis)
library(MASS)

## Install GitHub packages (only if not already installed)
if (!requireNamespace("refloraR", quietly = TRUE)) {
  devtools::install_github("DBOSlab/refloraR")
}
if (!requireNamespace("LCVP", quietly = TRUE)) {
  devtools::install_github("idiv-biodiversity/LCVP")
}
if (!requireNamespace("lcvplants", quietly = TRUE)) {
  devtools::install_github("idiv-biodiversity/lcvplants")
}
if (!requireNamespace("barRoso", quietly = TRUE)) {
  devtools::install_github("DBOSlab/barRoso", force = TRUE)
}

## Load GitHub packages
library(refloraR)
library(LCVP)
library(lcvplants)
library(barRoso)
```

## Load Pre-saved Reflora Records (Recommended)

To avoid long download times and possible knitting errors, we suggest
loading pre-downloaded `.rds` files instead of querying
Reflora during knitting.

```
# Load all Reflora records (filtered to Brazil)
records_br <- readRDS("records_reflora_br.rds")

# Load indeterminate specimens (filtered to Brazil)
indets_br <- readRDS("indets_reflora_br.rds")
```

### Optional: Download from Reflora (Not evaluated by default)

```
## downloading all records
records_reflora <- refloraR::reflora_records(
  save = FALSE
)

## standardizing retrieved records
records_reflora_std <- barRoso::barroso_std(
  records_reflora,
  unvouchered = TRUE,
  delunkcoll = TRUE,
  flag_missid = TRUE,
  flag_duplicates = TRUE,
  rm_duplicates = TRUE,
  colname_recordedBy = "recordedBy",
  colname_recordNumber = "recordNumber",
  colname_country = "country",
  colname_stateProvince = "stateProvince",
  colname_municipality = "municipality",
  colname_locality = "locality",
  colname_collectionCode = "collectionCode",
  colname_institutionCode = "institutionCode",
  colname_typeStatus = "typeStatus",
  colname_family = "family",
  colname_genus = "genus",
  colname_specificEpithet = "specificEpithet",
  rm_original_column = TRUE
)

## Brazilian records
records_br <- records_reflora_std %>% dplyr::filter(country == "Brazil")

## downloading all indeterminate specimens
indets_reflora <- refloraR::reflora_indets(save = FALSE)

## standardizing retrieved records
indets_reflora_std <- barRoso::barroso_std(
  indets_reflora,
  unvouchered = TRUE,
  delunkcoll = TRUE,
  flag_missid = TRUE,
  flag_duplicates = TRUE,
  rm_duplicates = TRUE,
  colname_recordedBy = "recordedBy",
  colname_recordNumber = "recordNumber",
  colname_country = "country",
  colname_stateProvince = "stateProvince",
  colname_municipality = "municipality",
  colname_locality = "locality",
  colname_collectionCode = "collectionCode",
  colname_institutionCode = "institutionCode",
  colname_typeStatus = "typeStatus",
  colname_family = "family",
  colname_genus = "genus",
  colname_specificEpithet = "specificEpithet",
  rm_original_column = TRUE
)


## Brazilian indeterminate species
indets_br <- indets_reflora_std %>% dplyr::filter(country == "Brazil") 

# Save for future use
saveRDS(records_br, "records_reflora_br.rds")
saveRDS(indets_br, "indets_reflora_br.rds")
```

# 1. Proportion of Indeterminate Records by Herbarium

## 1.1 Prepare Data

```
br_record_herb <- records_br %>%
                  count(collectionCode, name = "total")
```

## 1.2 Separate family and genus indet counts

```
#genera
br_indet_gen <- indets_br %>% filter(taxonRank == "GENUS") %>%
                              count(collectionCode, name = "indet_gen")
#families
br_indet_fam <- indets_br %>% filter(taxonRank == "FAMILY") %>% 
                              count(collectionCode, name = "indet_fam")
```

## 1.3 Merge and calculate proportions

```
df_combined <- br_record_herb %>%
             left_join(br_indet_gen, by = "collectionCode") %>%
             left_join(br_indet_fam, by = "collectionCode") %>%
                 mutate(across(c(indet_gen, indet_fam), ~replace_na(., 0)),
                        indet_total = indet_gen + indet_fam,
                        perc_indet_gen = 100 * indet_gen / total,
                        perc_indet_fam = 100 * indet_fam / total,
                        perc_indet_total = 100 * indet_total / total)
```

## 1.4 Top 25 herbaria by total specimens

```
plot_data <- df_combined %>%
             slice_max(order_by = indet_total,
                                        n = 25) %>%
             mutate(collectionCode = fct_reorder(collectionCode, total))
```

## 1.5 Pivot longer for plotting

```
stacked_df <- plot_data %>%
              pivot_longer(cols = c(indet_gen, indet_fam),
                  names_to = "level", values_to = "count") %>%
             mutate(
                   level = recode(level,
                   indet_gen = "Genus-level",
                   indet_fam = "Family-level"),
                   percentage = 100 * count / total)
```

## 1.6 Plotting

```
##principal plot
p_stack <- ggplot(stacked_df, aes(x = collectionCode,
                                  y = percentage, fill = level)) +
  geom_col(data = stacked_df %>% group_by(collectionCode) %>% slice(1),
           aes(y = 100), fill = "grey90", width = 0.8) +
  geom_col(width = 0.8) +
  geom_text(data = plot_data, aes(x = collectionCode, y = 104, label = total),
            size = 3.5, vjust = 0, inherit.aes = FALSE) +
  scale_fill_manual(values = c("Genus-level" = "#E69F00",
                               "Family-level" = "#56B4E9")) +
  labs(
    x = "Herbarium (Collection Code)",
    y = "% of Records",
    fill = "Indeterminate Level",
    title = "Indeterminate specimens in the Top 25 REFLORA Herbaria"
  ) +
  coord_flip() +
  theme_minimal(base_size = 12) +
  theme(
    axis.text.y = element_text(size = 10, face = "bold"),
    panel.grid.major.y = element_blank(),
    panel.grid.major.x = element_line(color = "grey90"),
    plot.title = element_text(size = 14, face = "bold"),
    legend.position = "right"
  )

##adding text
p_stack <- p_stack +
  geom_text(data = plot_data,
            aes(x = collectionCode,
                y = perc_indet_total + 3,
                label = indet_total),
            size = 3.2, vjust = 0, fontface = "bold",
            inherit.aes = FALSE)

##showing
p_stack
```

# 2. Map of Brazil showing the proportion of indeterminate specimens

## 2.1 Load Pre-saved cleaned data (Recommended)

To avoid long download times and possible knitting errors, we suggest
loading pre-downloaded `.rds` file instead of processing the
coordinate cleaner and other processes

```
# Load indeterminate records, in sf format 
indets_br_sf <- readRDS("indets_br_sf.rds")
```

## Optional. Process the geographical and other cleaning mechanisms

```
##factor 
indets_br$taxonRank <- factor(indets_br$taxonRank, 
                              levels = unique(indets_br$taxonRank),
                              ordered = TRUE)


##using scientific name for cleaning
indets_br <- indets_br %>%
  mutate(
    scientificName = ifelse(taxonRank == "GENUS", genus, family) 
    # Use genus or family name as placeholder
  )


clean_invalid_coordinates <- function(df) {
  # Coerce columns to numeric
  df$decimalLatitude <- as.numeric(df$decimalLatitude)
  df$decimalLongitude <- as.numeric(df$decimalLongitude)
  
  # Remove rows with NA
  df <- df[!is.na(df$decimalLatitude) & !is.na(df$decimalLongitude), ]
  
  # Filter to valid latitude and longitude ranges
  df <- df[
    df$decimalLatitude >= -90 & df$decimalLatitude <= 90 &
      df$decimalLongitude >= -180 & df$decimalLongitude <= 180,
  ]
  
  return(df)
}


indets_br <- clean_invalid_coordinates(indets_br)


##defining the flags from Coordinate Cleaner
flags <- clean_coordinates(x = indets_br,
                           lon = "decimalLongitude",
                           lat = "decimalLatitude",
                           species = "genus",
                           tests = c("capitals", "centroids", "gbif", 
                                     "institutions", "seas", "urban", "zeros"))


# Exclude problematic records
indets_br <- indets_br[flags$.summary,]


##transforming to sf
indets_br_sf <- indets_br %>%
  sf::st_as_sf(coords = c("decimalLongitude", "decimalLatitude"),
               crs = 4326)

saveRDS(indets_br_sf, "indets_br_sf.rds")
```

## 2.2 Create subset of indet records at family and genus level

```
#family
indets_br_sf_family <- indets_br_sf %>% filter(taxonRank == "FAMILY" & !is.na(family))
#genera
indets_br_sf_genus <- indets_br_sf %>% filter(taxonRank == "GENUS")
```

## 2.3 Brazilian map and contextualize indet data

```
##Brazilian map
brazil <- geobr::read_country(year = 2020, simplified = TRUE)
brazil <- sf::st_transform(brazil, crs = 4326)


## Joining for eliminating outside frontiers
indets_br_sf_genus <- sf::st_join(indets_br_sf_genus,
                                  brazil, join = st_within, left = FALSE)
indets_br_sf_family  <- sf::st_join(indets_br_sf_family, brazil,
                                    join = st_within, left = FALSE)
```

## 2.4 Plotting

```
## family plot
family_plot <- ggplot() +
  geom_sf(data = brazil, fill = "gray95", color = "gray60") +
  geom_sf(data = indets_br_sf_family, color = "#56B4E9", size = 1, 
          alpha = 0.5) +
  labs(title = "Indet. sp. Family Level") +
  theme_minimal() +
  theme(
    plot.title = element_text(size = 14, face = "bold"),
    plot.subtitle = element_text(size = 12),
    axis.text.x = element_text(size = 12),
    axis.text.y = element_text(size = 12)
  )


## genus plot
genus_plot <- ggplot() +
  geom_sf(data = brazil, fill = "gray95", color = "gray60") +
  geom_sf(data = indets_br_sf_genus, color = "#E69F00", size = 1, 
          alpha = 0.5) +
  labs(title = "Indet. sp. Genus Level") +
  theme_minimal() +
  theme(
    plot.title = element_text(size = 14, face = "bold"),
    plot.subtitle = element_text(size = 12),
    axis.text.x = element_text(size = 12),
    axis.text.y = element_text(size = 12)
  )


# Combine plots side by side
combined_plot <- family_plot + genus_plot +
  plot_layout(ncol = 2) & 
  theme(plot.margin = margin(10, 10, 10, 10))

##displaying
combined_plot
```

# 3. Map of Brazil displaying the density of indeterminate specimens

## 3.1 load Brazil shapefile and organize data

```
# Load Brazil shapefile
df_brazil <- ne_countries(scale = "medium", returnclass = "sf") %>%
  filter(admin == "Brazil")


# Create subsets of indet records at family and genus level
indets_br_family <- indets_br %>% filter(taxonRank == "FAMILY" & !is.na(family)
                                         &!is.na(decimalLatitude) &
                                         !is.na(decimalLongitude))
indets_br_genus <- indets_br %>% filter(taxonRank == "GENUS"
                                        &!is.na(decimalLatitude) &
                                         !is.na(decimalLongitude))
```

## 3.2 Family threatment

```
# I. Convert to sf object
points_sf <- sf::st_as_sf(indets_br_family, 
                          coords = c("decimalLongitude", "decimalLatitude"), 
                          crs = 4326)


# II. Spatial filter using Brazil polygon
points_sf <- sf::st_join(points_sf, df_brazil, join = st_within, left = FALSE)


# III. Hexbin map to show sampling effort (collection gaps)
hex_data <- st_coordinates(points_sf) %>%
  as.data.frame() %>%
  rename(lon = X, lat = Y)


# IV. Smooth raster-style density map (masked to Brazil, yellow emphasized)
kde <- with(hex_data, kde2d(lon, lat, n = 300, lims = c(-74, -34, -34, 6), h = c(0.5, 0.5)))
density_df <- as.data.frame(expand.grid(x = kde$x, y = kde$y))
density_df$layer <- as.vector(kde$z)


# V. Convert to sf and mask to Brazil
st_geometry(density_df) <- st_sfc(lapply(1:nrow(density_df), 
                          function(i) st_point(c(density_df$x[i],
                                                 density_df$y[i]))),
                                                            crs = 4326)
##density conversion
density_sf <- st_as_sf(density_df)
density_masked <- density_sf[df_brazil, ]
##masked coordinates
masked_coords <- st_coordinates(density_masked) %>%
                                          as.data.frame() %>%
                                       bind_cols(layer = density_masked$layer)

# VI. Family map
p_raster_family <- ggplot() +
  geom_sf(data = df_brazil, fill = viridis(1), colour = "gray40", size = 0.3) +
  geom_raster(data = masked_coords, aes(x = X, y = Y, fill = layer)) +
  geom_sf(data = df_brazil, fill = NA, colour = "gray40", size = 0.3) +
  coord_sf(xlim = c(-74, -34), ylim = c(-34, 6), expand = FALSE) +
  scale_fill_viridis_c("Specimen Density", option = "cividis",
                       trans = "sqrt",  direction = -1) +
  theme_void() +
  theme(legend.position = c(0.25, 0.25)) +
  labs(title = expression(bold("A) ") * "Indet. sp. (Family level)") )
```

## 3.3 Genera threatment

```
#I. Convert to sf object
points_sf <- sf::st_as_sf(indets_br_genus, 
                          coords = c("decimalLongitude", "decimalLatitude"), 
                          crs = 4326)


#II. Spatial filter using Brazil polygon
points_sf <- sf::st_join(points_sf, df_brazil, join = st_within, left = FALSE)


#III. Hexbin map to show sampling effort (collection gaps)
hex_data <- st_coordinates(points_sf) %>%
                    as.data.frame() %>%
                    rename(lon = X, lat = Y)

#IV. Smooth raster-style density map (masked to Brazil, yellow emphasized)
kde <- with(hex_data, kde2d(lon, lat, n = 300, lims = c(-74, -34, -34, 6),
                            h = c(0.5, 0.5)))
density_df <- as.data.frame(expand.grid(x = kde$x, y = kde$y))
density_df$layer <- as.vector(kde$z)


#V. Convert to sf and mask to Brazil
st_geometry(density_df) <- st_sfc(lapply(1:nrow(density_df),
                                         function(i) st_point(c(density_df$x[i],
                                              density_df$y[i]))), crs = 4326)
density_sf <- st_as_sf(density_df)
density_masked <- density_sf[df_brazil, ]


masked_coords <- st_coordinates(density_masked) %>%
  as.data.frame() %>%
  bind_cols(layer = density_masked$layer)


#VI. Plot density map
p_raster_genus <- ggplot() +
        geom_sf(data = df_brazil,
                fill = viridis(1), colour = "gray40", size = 0.3) +
  geom_raster(data = masked_coords, aes(x = X, y = Y, fill = layer)) +
  geom_sf(data = df_brazil, fill = NA, colour = "gray40", size = 0.3) +
  coord_sf(xlim = c(-74, -34), ylim = c(-34, 6), expand = FALSE) +
  scale_fill_viridis_c("Specimen Density", option = "cividis", trans = "sqrt",  direction = -1) +
  theme_void() +
  theme(legend.position = c(0.25, 0.25)) +
  labs(title = expression(bold("B) ") * "Indet. sp. (Genus level)") )
```

## 3.4 Combining plots

```
combined_plot <- p_raster_family + p_raster_genus +
  plot_layout(ncol = 2) & 
  theme(plot.margin = margin(10, 10, 10, 10))

##displaying
combined_plot
```
